# A Service-Oriented Middleware for Integrated Management of Crowdsourced and Sensor Data Streams in Disaster Management †

**DOI:** 10.3390/s18061689

**Published:** 2018-05-24

**Authors:** Luiz Fernando F. G. de Assis, Flávio E. A. Horita, Edison P. de Freitas, Jó Ueyama, João Porto de Albuquerque

**Affiliations:** 1 Institute of Mathematics and Computer Science (ICMC), University of São Paulo (USP), São Carlos/SP 13566-590, Brazil; flavio.horita@ufabc.edu.br (F.E.A.H.) joueyama@icmc.usp.br (J.U.); J.Porto@warwick.ac.uk (J.P.d.A.); 2 Federal University of Rio Grande do Sul, Porto Alegre/RS 90040-060, Brazil; edison.pignaton@inf.ufrgs.br; 3 Centre for Interdisciplinary Methodologies, University of Warwick, Coventry CV4 7AL, UK; 4 Center for Mathematics, Computation and Cognition, Federal University of ABC, Santo André/SP 09210-580, Brazil

**Keywords:** Service-Oriented Middleware, Sensor Web Enablement, Disaster Management, Big Data Stream, Crowdsourcing

## Abstract

The increasing number of sensors used in diverse applications has provided a massive number of continuous, unbounded, rapid data and requires the management of distinct protocols, interfaces and intermittent connections. As traditional sensor networks are error-prone and difficult to maintain, the study highlights the emerging role of “citizens as sensors” as a complementary data source to increase public awareness. To this end, an interoperable, reusable middleware for managing spatial, temporal, and thematic data using Sensor Web Enablement initiative services and a processing engine was designed, implemented, and deployed. The study found that its approach provided effective sensor data-stream access, publication, and filtering in dynamic scenarios such as disaster management, as well as it enables batch and stream management integration. Also, an interoperability analytics testing of a flood citizen observatory highlighted even variable data such as those provided by the crowd can be integrated with sensor data stream. Our approach, thus, offers a mean to improve near-real-time applications.

## 1. Introduction

Public interest in measuring various conditions with low cost devices has increased in remidrulcent years. Big data streams provided by sensors and the Internet of Things have emerged as an alternative source of information for highly diverse applications, such as e-agriculture, smart cities, and disaster management. They entail dynamic, intermittent management of sensors with distinct protocols and interfaces, which provide a massive number of heterogeneous, continuous, and unbounded data streams at a rapid rate. In sustainable agriculture, for example, farmers have attached sensors to animals to act as mobile sensors. Data thus generated could mitigate crop issues such as pest and water stress in near-real time. Farmers also use microdrones to fertilize crops saving time, money, and fertilizer. In other applications, such as disaster management, decision makers have used similar initiatives to provide current data to enhance prediction, as well as to take more informative and proper decisions [1].

In 2016, natural disasters occurred in considerably great numbers than the average annual rates (https://www.munichre.com/topics-online/en/2017/topics-geo/overview-natural-catastrophe-2016) for the last 10 years and 30 years (590 and 470, respectively) resulting in more than US$175 billion worth of loss and damage. Hydrological events (floods, flash floods, mass movements) reached 50% of natural disasters worldwide, with the USA, Europe, China as major contributors. Floods account for 18% of the insured loss, values subtracted from the overall loss. Floods in France and severe flash floods in Germany and Austria (https://www.theguardian.com/world/2016/jun/02/deaths-as-flash-floods-hit-france-germany-and-austria) highlight that even well prepared countries need measures to manage disaster risks effectively and efficiently and reduce their impact on human settlements, economic assets, cultural heritage, social infrastructure [2].

Federal water agencies (http://www.ana.gov.br/PortalSuporte/frmSelecaoEstacao.aspx) (https://www.pegelonline.wsv.de/gast/pegeltabelle) promote historical stationary sensor data categorized by measurement thresholds to enhance community resilience. Deploying an increasing number of sensors for environmental monitoring with a distributed discrete setting, however, is inadequate to meet community needs. Stationary sensors cannot provide an overview of as wide an area as mobile ones. Mobile sensors, such as unmanned aircraft systems and satellites, have operated at heights and distances that manned system cannot do with as safely [3,4,5]. Such devices combine control stations, communication links, and sensors for flights of extended duration. In view of the difficulty of their maintenance and propensity for error, decision makers have considered supplementing their data with timely information provided voluntarily by residents in hazardous areas [6]. Such “citizen as sensors” [7,8] are part of a body of individuals who respond to local needs and comprise a network with over 7.5 billion nodes capable of synthesizing and interpreting local data using their senses and intelligence.

Since they can provide miscellaneous, georeferenced observations, they are referred herein as heterogeneous geosensors, and their potential has been noted in several recent studies. None of the present approaches [9,10,11,12,13], however, indicates how to access, filter, and publish data in an interoperable, flexible manner that facilitates rapid decision making. To achieve this requires addressing the following issues: (i) the wide variety of geosensor protocols and interfaces [14,15]; (ii) the fact that most of the applications rely on their own mechanisms to perform geosensor management [16]; (iii) the coupling of a set of services required for providing, processing and storing heterogenous geosensor data [17]; and (iv) the dearth of near-real-time geosensor data-stream processing given filtering applications’ parameters [18].

In attempt to limit the effects of the idiosyncrasies in implementing heterogeneous geosensors on applications, the Open Geospatial Consortium launched a Sensor Web Enablement (SWE) initiative. The initiative defines rules and guidelines for the interoperability of sensors connected to the Web [19,20]. Although it provides a set of operations to find, extract and publish heterogeneous geosensors data for managing data batches [21,22]. This is not the sole management need in situations in which sensors are continuously embedded and removed, a common practice in the management of floods and other dynamic disasters.

The prevention and mitigation of disasters are intrinsically related to complex management issues. Challenges range from sensors lacking battery power to monitoring an extensive area. The main assumption undelying disaster management is that nothing should be taken for granted. Instead, there is an ad hoc network with distinct capacity sensors monitoring events. Sensors amass and transmit numerous heterogeneous data flow rates and formats. As a result, it can become difficult to find geosensor data streams based on the three principal filtering parameters: spatial, temporal and thematic, a useful feature when users are looking for specific data such as water level and pressure or, in more complex cases, data from geosensors in distinct regions at different moments.

The ensemble of previously noted issues motivated the authors to design a generic, open, reusable approach to bridge the gap between low-level geosensor functionality management and application deployment by developing a middleware that enables interoperable communication between geosensors and applications.

This study extends the authors’ previous research: In Assis et al. [22], a module to extend Sensor Web, complying with SWE standards, is presented that facilitates integration of heterogeneous geosensors involved in scenarios characterized by constant change. While in Assis et al. [11], an experimental evaluation with qualitative and quantitative data is described that prioritizes in near-real-time location-based social network messages based on heterogeneous sensor data streams. In this study, the authors reinforce their prior findings and report on the following innovations:

1. **Service-Oriented Middleware.** The design, implementation, and evaluation of a service-oriented middleware for heterogeneous geosensor data to support on-the-fly access, near-real-time publication, and event filtering capabilities.
2. **Joining batch and streaming processing.** The pairing of batch and stream platforms to manage geosensors in dynamic scenarios, using generic, open and reusable components under the Sensor Web standards, and a general streaming engine for big data processing.
3. **Case study.** The leassons learned from the real-world application of flood risk management in Brazil.

The study is organized as follows. Section 2 outlines its background and principal concepts as described in the literature. Section 3 describes the motivation to develop the middleware in terms of specific requirements, and Section 4 details the proposed approach. Section 5 presents an analysis and evaluation of the scenarios tested. Section 6 includes a discussion about the study’s findings, while Section 7 summarizes the conclusion and provides recommendations for further research.

## 2. Related Works

As a result of advances in digital electronics and wireless communications, wireless sensor networks (WSNs) have become practical. Base stations, sensor fields, and nodes (sensors, processors, batteries, and radio transmitters) consitute the principal parts of WSNs. Together, they allow decision makers to monitor a broad spectrum of environmental conditions such as temperature, humidity and pressure [23]. WSNs must address fault tolerance, scalability, deployment costs, hardware restrictions, topology, environment and energy consumption, among other factors, which approach distinct Open System Interconnection model layers (application, transport, network, data link and physical), configurations (e.g., random networks) and mobilities (stationary and mobile sensors) [24]. Heterogeneous WSNs can operate at greater distances for longer durations than manned systems and detect missing data from unstable and problematic areas [3,25]. Their hardware diversity results in different data formats, flows and rate publication [26].

In addition to such traditional heterogeneous WSNs, the authors have considered a network using human volunteers as sensors. These “citizens as sensors,” equipped with intellect and senses, reflect and interpret what they observe [7]. They comprise more than 7.5 billion components that summarize local data. Over the years, people with different education histories have played roles analogous to those executed by government agencies. Such activities frequently involve volunteers but sometimes fail to attain accurate results. Volunteers have greater impact on geographic information systems in collectively providing volunteered geographic information (VGI). Through crowdsourcing platforms such as OpenStreetMap (http://www.openstreetmap.org/), Ushahidi (http://www.ushahidi.com/), Wikimapia (http://wikimapia.org) and citizens observatories (https://cobwebproject.eu/), they gather and transmit data to an uplink node, using Web 2.0, geo-referencing, geo-tags, GPS and broadband communication.

Thus, traditional and citizen sensors perform complementary tasks as a macro-instrument suitable for environmental monitoring and sensor exploration [27]. Both interact with a synergy analogous to that of neurons within the brain [28,29]. Applied as the macro-instrument Sensor Web, they can enable warning systems to predict, estimate and monitor natural hazards [30]. Besides identifying sensor networks available online, Sensor Web designates an intermediate layer to bridge the gap between sensor network low-level components and applications, acting as middleware it masks hardware components complexity and heterogeneity from local users [31].

Middleware mitigates heterogeneous sensor challenges, in particular those related to limited resources, and enables applications to locate dynamic network sensors and services, thus enhancing system resource platform management and adding predictability to applications [32]. Its development requires addressing not only sensor network power management but also scalability, mobility, heterogeneity, usability and transparency to use memory and process power transmission switching more efficiently to accomodate dynamic changes in network size. Thus, middleware renders complex heterogeneous abstract models accessible through a user-friendly application program interface [33].

Most current middleware only integrates sensors with their own mechanisms [34,35,36,37,38]. Although some implementations seek to become generic and abstract, it is unfeasible to find reusable components. A sensor abstract layer provides a plug-in model, in which applications can load new types of sensors on an operating system. However, this approach like other middleware solutions [39,40,41,42,43,44] fails to consider available open standards [45].

SWE-based middleware, on the other hand, establishes transparent communication between sensors and Web applications and incorporates a set of standard activities to discover, exchange, and process sensor data [46]. Several applications rely on standards such as SWE and device profile for Web Services to integrate sensors, but they fail to manage the context involved in publishing data to the Web in near real-time [18,47,48,49,50,51]. To mitigate this challenge, the message bus architecture Sensor Bus comprises a common communication infrastructure, a set of adaptable interfaces and a well-defined protocol and provides semantically-enabled sensor plug-and-play via an automatic mediation between semantic sensors and SWE standards [52,53,54,55,56]. Sensor Bus, however, does not use observation capability metadata to improve data discovery [57] nor does it simplify data access or mask the complexity of SWE standards [21]. Moreover, it lacks the ability to manage data streams in light of spatial, temporal and thematic contexts [58].

The challenge remains to provide functions integrated with complex, large-scale processing capabilities in an event-driven manner [59,60]. A publish/subscribe approach could define ways to meet these needs [61], but scientists are just catching a glimpse of event-stream processing. Accordingly, filtering occurs without enterprise service bus, complex event processing and event pattern markup language, which facilitate the identification of relevant events in an interoperable approach [62]. Furthermore, the absence of an information broker such as Web Notification Service preclude forwarding relevant broadcast notifications to the applications [63].

Integrating complex processing units into synchronized data streams to manage dynamic environmental data provided by traditional and citizen sensors in near real-time remains critical. Adopting a generic approach based on SWE standards [14,64] can overcome, to some degree, the obstacles posed by diverse sensor specifications and implementations. This approach will facilitate risk management and impact reduction by providing a fast, integrated, and reliable response time [65]. This complex, cyclical task involves specific activities before, during and after critical dynamic situations such as floods [66], which endanger human lives and property.

## 3. Middleware Requirements for Dynamic Scenarios

The middleware requirements for dynamic scenarios derived from a systematic mapping review [67] of the related works presented in the previous section are presented in this one, which brings the research up to date with recent studies. These requirements correspond to the functions needed to manage sensors, Sensor Web services and applications. The intent is to guide scientists from designing the context in which the middleware will operate to prioritizing its functions and behaviors, while facilitating data organization. Keeping these necessities in mind, their specification may be viewed from two distinct perspectives: sensors and applications.

Given the significant variety of templates to represent a single requirement specification, the study focus on summarizing the principal functions provided by current middleware (see Table 1), which facilitates validation of the study’s methodology. As a consequence, the set of functions that the middleware proposed herein should provide comprise sensor and service registration and updating, data publication, access to near-real-time and historical sensor data, access to service and sensor metadata, sensor and service subscription, sensor selection and tasking, and semantic correspondence of sensors and services.

First, from a sensor perspective, middleware enables sensor and service data publication, which includes automatic self-registration of sensors and services on the Web as long as they remain active and their updating, as needed. Data publication is a critical task and includes sensor advertising in scenarios involving applications notification by publish/subscribe sensors. Then, from a service perspective, it is imperative that repositories store heterogeneous, historical, geo-referenced data without affecting near-real-time requests sent by diverse geo-sensors specifications and encoded in an intelligible, machine-readable format. These functions require components that prioritize scalability and adaptability.

Metadata access facilitates the integration of different interfaces, limiting coupling between components, removing their dependence on layers, and encapsulating the sensor and service functionalities without impacting applications. This interoperability guarantees reuse, adaptation and composition of components implemented in the middleware.

**Figure 1:**
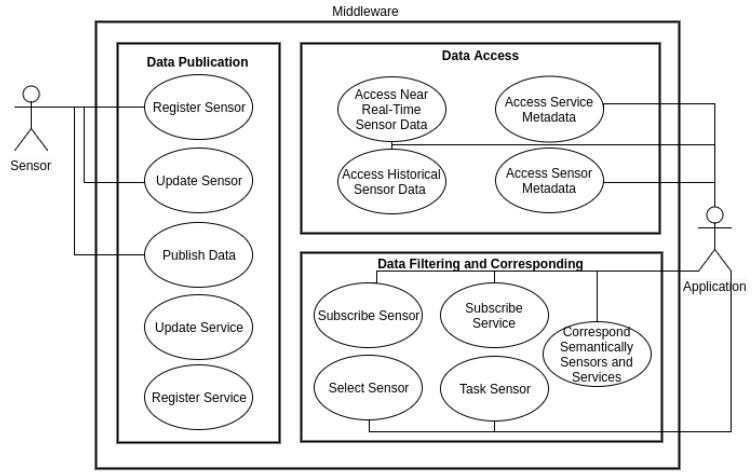
Use Cases Diagram—Middleware requirements.

Applications require services that receive and forward requests to the appropriate parties by means of a common interface that mitigates the complexity of parsing and encoding requests that add or remove components and enables contextual selection and categorization of data. For example, applications select specific sensors based on thematic, spatial and temporal parameters, by receiving data in a push-based or other manner without requesting it. In sum, semantic correspondence between sensors and services can improve an automatic matchmaking despite their diversity. A use case diagram of these requirements is provided in Figure 1.

## 4. AGORA—DSM

**Figure 2:**
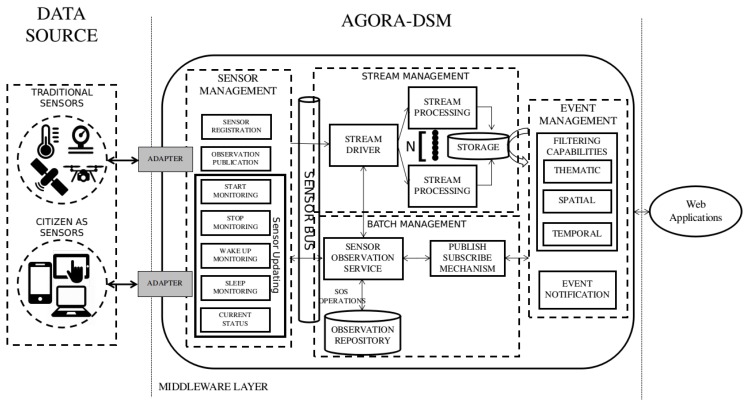
AGORA-DSM architecture.

This section presents the proposed service-oriented middleware for large-scale geosensor data-stream management (AGORA-DSM). The middleware enhances communication between geosensors and applications via open standard protocols and stream processing engines and provides publication of dynamic geo-sensor data in near real-time. It also offers data filtering, using thematic, spatial, and temporal parameters through data management that approaches Sensor Web services and scalable, high-throughput, fault-tolerant stream processing. To this end, its employs source adapters and sensor (SM), stream (StrM), batch (BM), and event management (EM) components (see Figure 2). This architecture was modeled using an informal notation language because current ones do not offer a unified model that provides the structure and operations of system and subsystem components.

### 4.1. Adapters

Adapters interpret data source formats and convert them into messages that middleware can process. The messages are part of a protocol that helps translate sensor status into sensor activities in dynamic situations. These tasks encompass registering new sensors, publishing data, and updating sensors. This approach extends that presented in [22] to improve communication between heterogeneous geosensors, including citizen sensors (Table 2) and is built on this protocol [52] due to its consistent and lightweight approach to managing sensor activities described in Section 4.3. The messages also facilitate ad hoc StrM inquiries. The enhanced version defines which properties sensors measure in the register sensor and the location field within sleep monitoring and stop monitoring tasks.

The first message registers new sensors and makes them acessible on the Web. This registering message contains the sensor’s identifier and the property it measures. Subsequently, the sensor can publish observations, i.e., acts of collecting data such as temperature, pressure or luminosity, represented by numerical, textual, or binary values. This message contains the sensor’s identifier, and the observation’s time, location and value. Sensors may also have their information updated. This message is useful when mobile sensors have distinct latitudinal, longitudinal positions and active and passive states over time, such as when a sensors starts and stops monitoring an area and sleeps or wakes from a maintenance process during execution of a task, fields separated by an asterisk.

### 4.2. Sensor Management (SM)

**Figure 3:**
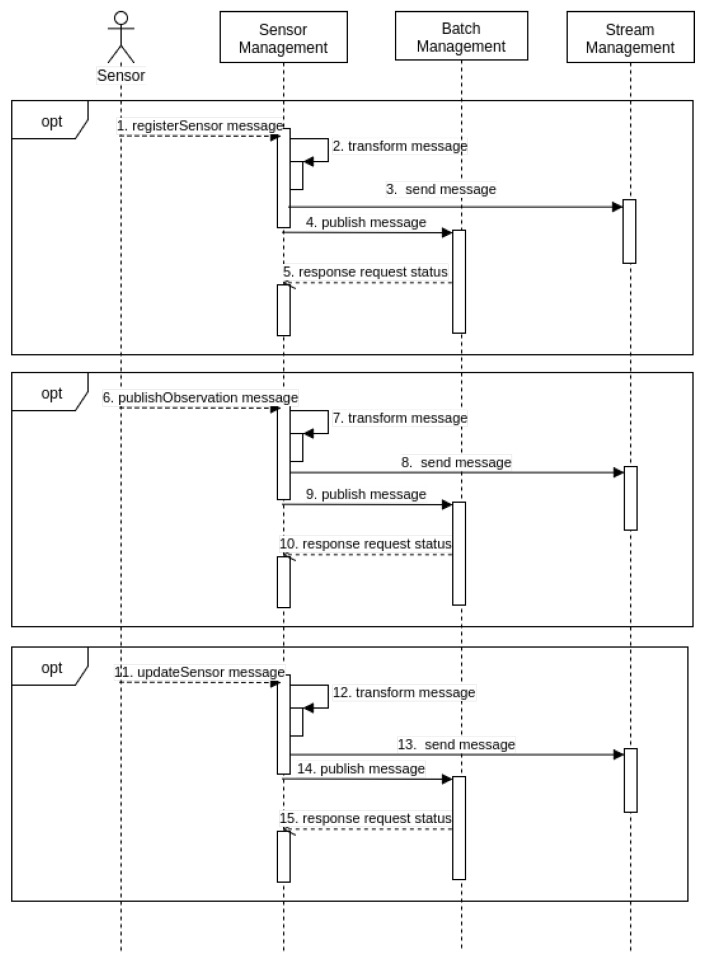
Interactions between Sensors, SM, BM and StrM arranged in time sequence.

Sensor management involves incoming messages already transformed into sensor activities of their respective batch and stream management operations. SM also manages the following sensor activities: sensor registration, observation publication and sensor updating (see Figure 3). Its purpose is to enhance interoperability in situations involving a broad range of sensor specifications and implementations. SM and its adapters usually run on a computing unit of the sensor gateway, preserving system resources and containing configuration BM and StrM parameters.

#### 4.2.1. Sensor Registration

When a sensor communicates with the middleware for the first time, it sends a registration message. This is necessary as the middleware does not store observations from unregistered sensors. The adapters convert this message into sensor registration activity, and the related component verifies the sensor by transmitting operations to the BM and StrM components.

#### 4.2.2. Observation Publication

Once registered, sensors are able to publish observations as the adapters convert sensor data into observation publication activity, and the relevant component parses the message and translates it into an observation using BM and StrM operations.

#### 4.2.3. Sensor Updating

As a result of extensive mobility, uncovered areas, and mechanical problems sensors frequently change their status, including position and activity. For example, mobile sensors change their positions when monitoring an area during a disaster. Subsequently, when their battery power is low, they cease monitoring. Accordingly, sensors publish on the Web when they stop sleeping (sleep monitoring) and when they resume it (wake up monitoring).

Finally, during flight, mobile aerial sensors keep their current position up to date on the Web, enabling stakeholders to consider new monitoring tracks, for example. It is essential to keep sensor status up to date because critical applications rely on near-real-time data. Accordingly, the components of the proposed approach are intended to improve the interoperability and integration of the heterogeneous sensors used to monitor dynamic conditions.

### 4.3. Batch Management (BM)

A sensor observation service (SOS) offers an interoperable interface to discover, manage, and recover near-real-time or historical sensor data. SOS defines a common model for sensor domain clients encapsulated by parameters of SOS operations. These parameters involve time (phenomenon and result time), procedures, observed properties, feature of interest and sensor response format (result and unit of measure). Thus, users may request sensor data using a variety of protocols.

**Figure 4:**
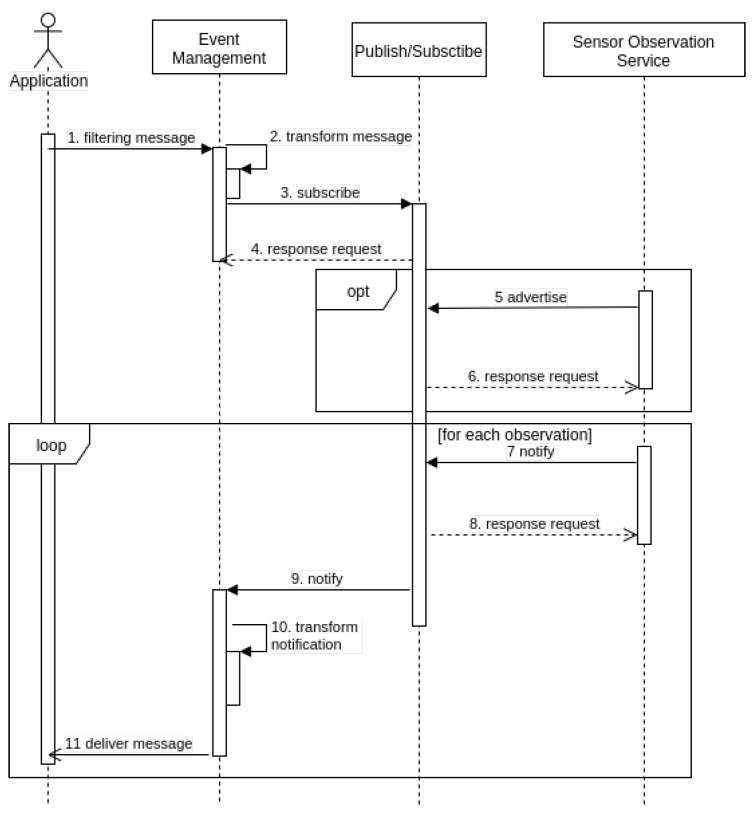
BM: Application, EM, Publish/Subscribe and SOS.

In the middleware, publish/subscribe acts as a broker that forwards data from the producer (SOS) to the consumers (Web application). Figure 4 depicts a scenario in which sensors transmit messages that describe events to the applications based on filter criteria. This is a role analogous to a request/reply messaging component, the difference being that the study’s publish/subscribe component enables ongoing, persistent, asynchronous expression.

### 4.4. Stream Management (StrM)

**Figure 5:**
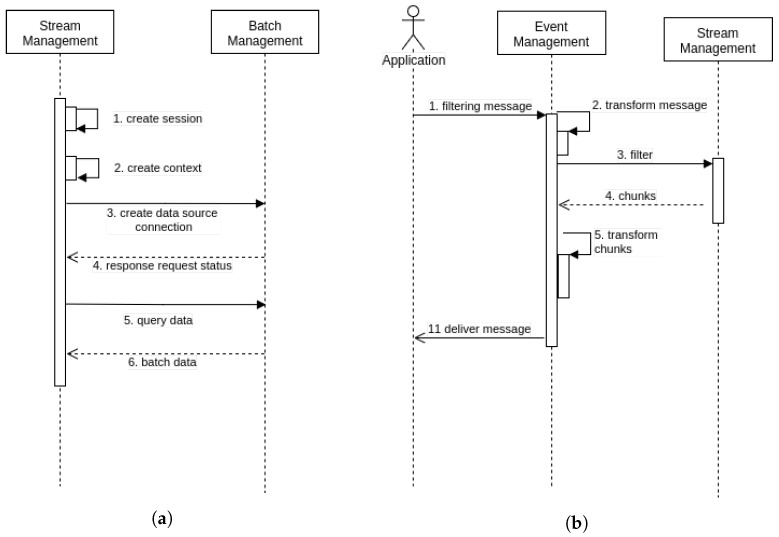
Interactions arranged in time sequence. (**a**) StrM and BM workflow; (**b**) Application, EM and StrM workflow.

Stream management enables reading a set of elements in a scalable, parallel, fault-tolerant manner, while limiting bandwidth and other resource network overhead. It operates ad hoc queries in memory by means of immutable, partitioned data as blocks of elements across a cluster of *N* machines, where *N* ≥ 1. These features help track data processing. Stream management may collect data from batch input sources or near-real-time providers (see Figure 5a,b). Then it converts the streaming data into micro-batches based on a set of configuration parameters. StrM comprises two main units: the stream driver and stream processing. The stream driver manages cluster resources, while stream processing operates datasets transformations and actions and prepares the output for storage.

### 4.5. Event Management (EM)

Event management parses and translates filtered messages sent and received by applications into BM and StrM components. EM comprises two principal functions: thematic, spatial and temporal filtering capabilities and event notification.

#### 4.5.1. Filtering Capabilities

Filtering capabilities mask the implementation details of selecting notifications based on filtering criteria submitted by applications. Filtering improves the management of disasters as it can provide observations from sensors deployed within a vulnerable area in a specific time period and transmitting particular data such as water level. For example, temporal, spatial, and thematic filtering capabilities can provide the number of times specific properties monitored by sensors within a given area exceed an established threshold during a particular interval as they can filter either all or part of incoming sensor data. Filters depend on sensor observation, and event management converts application parameters into messages and tests them, using different portions of data and units of measurement.

**Figure 6:**
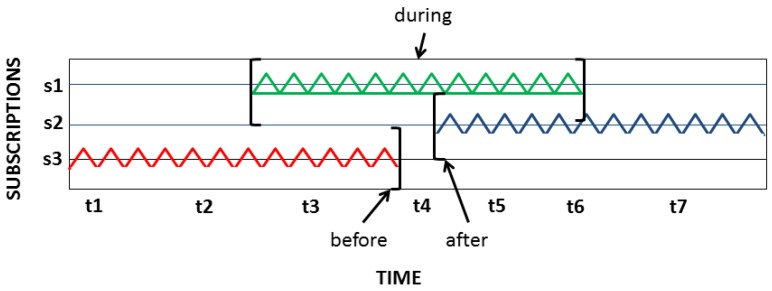
Filtering sensors using time as a parameter.

**Temporal Filtering:** Temporal filtering encodes ISO 19108:2002 and helps determine whether time parameters satisfy the message’s syntax and semantics. Temporal operators, such as *after*, *before*, *begin*, *during*, *end* and *equals*, take into account the temporal reference system. For instance, *start* and *end* time fields vary, depending on circumstances, while the operators *before*, *begin* and *after* require only one of these fields. Figure 6 depicts three filtering types: *s1* requires observations between an initial (*start*) and an *end* time (*during*); *s2* requires observations following an initial time (*after*); and, s3 requires observations preceding an *end* time (*before*).

**Figure 7:**
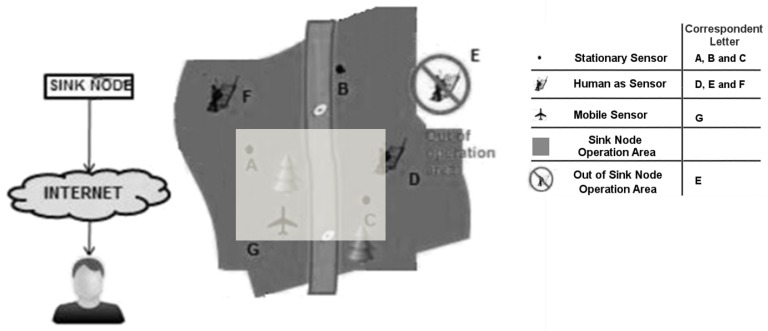
Filtering sensors using a bounding box as a paramater.

**Spatial Filtering:** Spatial filtering encodes ISO 19107:2003 and requires a correct syntax and semantic with geometries following a spatial reference system. Here the middleware determines whether the geometry of the sensor observation matches the geometrical pattern established by the application. The spatial filter comprises a point, a line, or a bounding box, and spatial operators focus on finding sensor observations within a given area. As can be seen in Figure 7, which depicts stationary, mobile sensors, and citizens sensors, only three heterogeneous are inside the bounding box.

**Figure 8:**
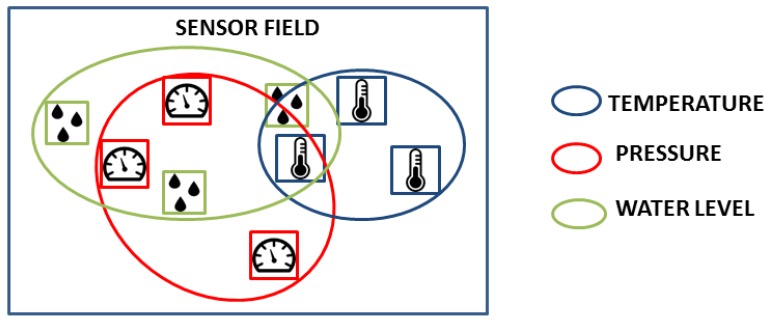
Filtering sensors using the property they provide.

**Thematic Filtering:** Thematic filtering imposes constraints on sensor observations in regard to the observed property. Figure 8 depicts three groups of nine sensors, measuring water level, pressure and temperature. Thematic filtering is critical in environmental monitoring in assessing the status of a specific disaster such as a flood. Images play a key role in such filtering as they provide an area overview that can inform scientists’ selection of the relevant properties to monitor.

#### 4.5.2. Event Notification

Following filtering, the middleware forwards appropriate notifications to applications of incoming sensor data based on established filters. The filters consider the diverse measurement units and enable applications to combine more than one filter type. For example, the middleware can provide mechanisms for applications to search for sensors that measure a temperature higher than 35 degrees Celsius in Rio de Janeiro, Brazil, during December 2017.

## 5. Experimental Evaluation

In this section, the run-time environment in which the middleware was used and quality measurements to assess sensor management were subsequently described. The study focuses on six measures: time behaviour, scalability, resource utilization, frequency, payload, and interoperability. An architectural analysis assessed the integration of batches with streams data and the use of the middleware to identify near-real-time events.

### 5.1. Study case: Flash floods in Brazil

The scenario of flash floods in Brazil is particularly concerning as it is anticipated to grow in frequency due to climate change. To cope with the impact of such catastrophes, the Brazilian government established the National Center for Monitoring and Early Warning of Natural Disasters (http://www.cemaden.gov.br/) (CEMADEN, in Portuguese), which issues advance warnings to relief organizations. The Civil Defense and Disaster Management Center uses more than 4000 hydrological and rainfall stations to monitor some 957 municipalities. Rainfall gauges provide volume in 60-min intervals time scale when it is not raining. When rain starts, they begin providing data hourly. The study uses this data, accessed through an online service that provides structured JavaScript Object Notation (JSON) files with ten fields: station code, station name, latitude and longitude, unit of federation, city name, state acronym, water level, data type, and time.

While data provided by rainfall gauges are useful in disaster management, remote sensing is an alternative and supplementary data source as it can indicate the environmental condition of an entire region. The study used remote sensing imagery provided by CLIMATEMPO (http://www.climatempo.com.br), a Brazilian company that monitors and forecasts meteorological conditions and provides imagery from Geostationary Operational Environmental Satellite-16 in different spatial resolutions through a Web Map Service. The study used an infrared image covering 2 km overlaid on a map of colored terrains.

### 5.2. Experimental Setup Scenarios

The middleware was implemented using an oriented-object programming language (Java), by means of a servlet container (Apache Tomcat 8) and a database management system extended by a geographic plug-in (PostgreSQL/PostGIS). The study used the 52 North 4.1 version implementation of the SOS 2.0 specification (https://github.com/52North/SOS), while the sensors and observation metadata considered was *SensorML 2.0* and *O&M 2.0* specification. Unfortunately, we could not evaluate publish/subscribe mechanism due to its lack of current implementations. The advantages of SOS 2.0 52 North implementation is that it uses open source code, which is easily adapted, and provides the service operations required for this study. Spark streaming (https://spark.apache.org/streaming/) was used as it does not require a standard processing model, such as MapReduce, in Hadoop streaming (https://hadoop.apache.org/docs/r1.2.1/streaming.html). Four scenarios (**1.** 2017-11-29 08:50:42–2017-11-30 12:15:16; **2.** 2017-12-12 07:57:56–2017-12-13 06:49:20; **3.** 2017-12-14 01:06:54–2017-12-15 12:24:45; **4.** 2017-12-16 08:18:04–2017-12-18 07:54:04) were run on periods of floods.

### 5.3. Performance Efficiency of Sensor Management

#### 5.3.1. Time Behaviour and Scalability

**Figure 9:**
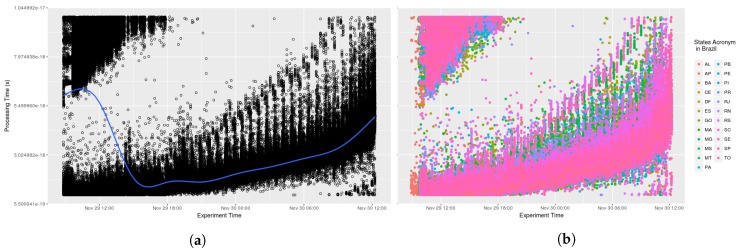
Scenario 01—Processing Time. (**a**) Stations in the whole country of Brazil; (**b**) Stations grouped by states.

**Figure 10:**
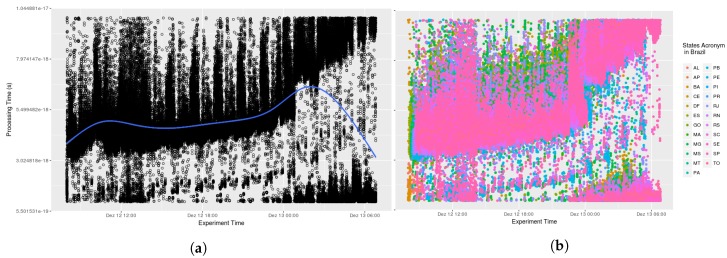
Scenario 02—Processing Time. (**a**) Stations in the whole country of Brazil; (**b**) Stations grouped by states.

**Figure 11:**
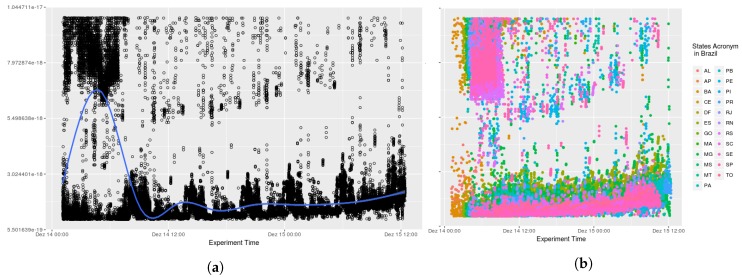
Scenario 03—Processing Time. (**a**) Stations in the whole country of Brazil; (**b**) Stations grouped by states.

**Figure 12:**
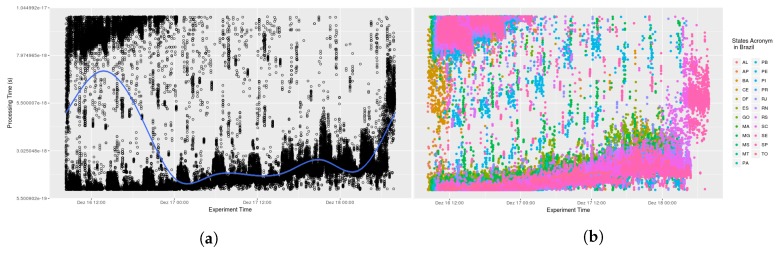
Scenario 04—Processing Time. (**a**) Stations in the whole country of Brazil; (**b**) Stations grouped by states.

Testing AGORA-DSM’s time behaviour and scalability involved analyzing the processing time required to collect stationary sensor data from CEMADEN. Initially, the time to receive, interpret and publish sensor data contained in the message was calculated. The data processing latency of 52 North implementations and Spark Streaming were not included as assessing their time behavior was outside the study’s scope. Thus, initial evaluations comprised the processing time to register sensors at their first observation and to publish their data, which are combined in the sensor API. Analysis found no significant impact arising from the diverse number of geosensors. The processing time took less than one second, an acceptable time for flood risk management. In Figure 9, it can be seen that at its onset messages took additional than subsequently in nearly all states as the database lacked a cache of the queries. Thus, time processing decreases across the initial hours (see Figure 10). This is evident when a smooth data line is generated using generalized additive models (GAM). Subsequently, AGORA-DSM processing time increases as a result of the increment in sensor data (see Figure 9 and Figure 10). This could be occasioned by floods, which increase the number of sensor data, or anomalies related to high values. Figure 11 and Figure 12 depict a scenario with data related to flood occurrence.

#### 5.3.2. Resource Utilization

**Figure 13:**
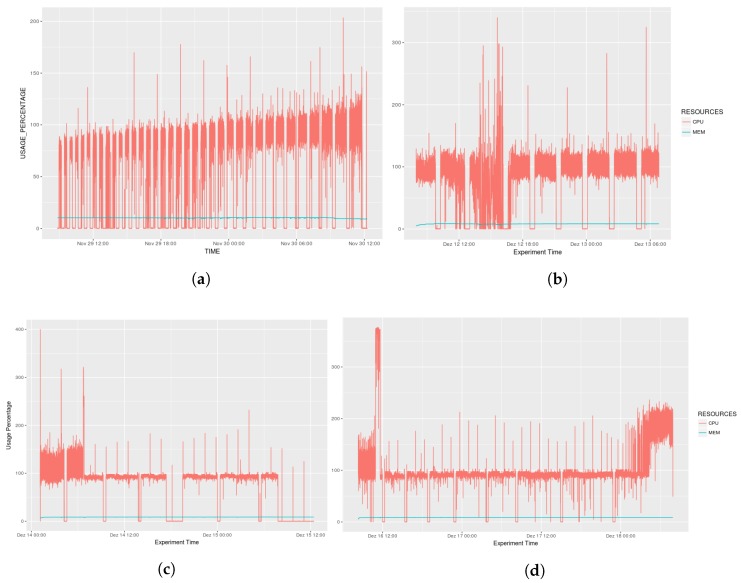
Resource Management. (**a**) Scenario 01; (**b**) Scenario 02; (**c**) Scenario 03; (**d**) Scenario 04.

AGORA-DSM’s use of computer resources, including CPU and Memory usage, in each scenario was evaluated during the extraction, transformation and loading of sensor data into the observation repository calculated (see Figure 13). Requesting data after full cycle of sensor data acquisition was terminated to mitigate potential overheads. Thus, CPU utilization presents a varied wave, while memory use is relatively constant. During execution, peaks of CPU utilization can be seen in Figure 13a,b, which also depicts variations as a result of overheated components slowing processing. Figure 13c,d show decreased fluctuations in CPU utilization as a result of decreased data.

#### 5.3.3. Requests Time Frequency

**Figure 14:**
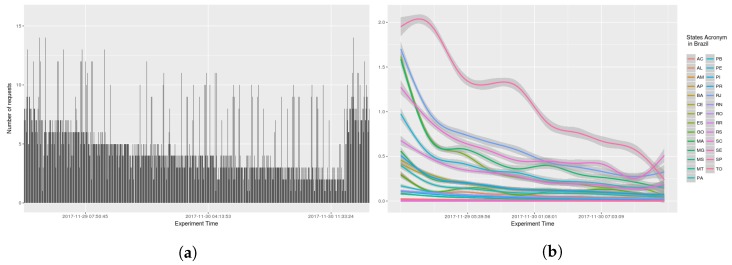
Scenario 01—Time Frequency. (**a**) Stations in the whole country of Brazil; (**b**) Stations grouped by states.

**Figure 15:**
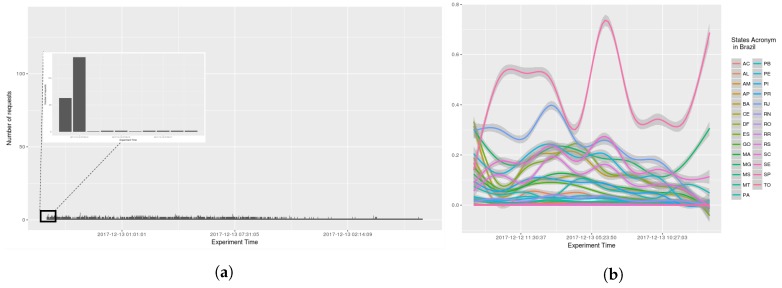
Scenario 02—Time Frequency. (**a**) Stations in the whole country of Brazil; (**b**) Stations grouped by states.

**Figure 16:**
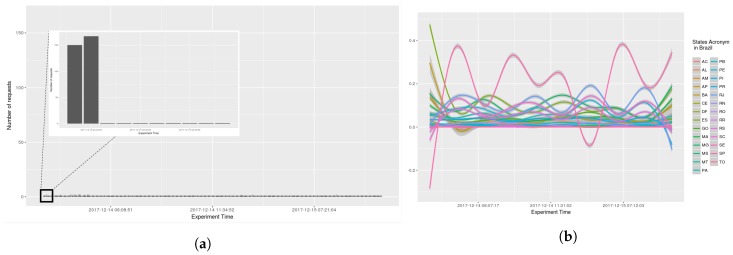
Scenario 03—Time Frequency. (**a**) Stations in the whole country of Brazil; (**b**) Stations grouped by states.

**Figure 17:**
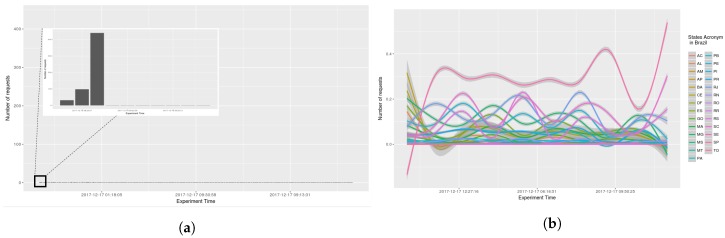
Scenario 04—Time Frequency. (**a**) Stations in the whole country of Brazil; (**b**) Stations grouped by states.

The number of requests per seconds received by AGORA-DSM, was examined (see Figure 14, Figure 15, Figure 16 and Figure 17). At the onset, bursts of requests were in all the scenarios, but subsequently they stabilize at less than 10 requests per second. GAM was reapplied with 0.95 confidence level, indicating a trend, i.e., because values are sometimes 0, GAM presents negative values in analysis by states (see Figure 15b and Figure 16b).

#### 5.3.4. Payload Size

The protocol proposed is simple and lightweight and could complement SWE Standards at a low cost from a lexical analysis perspective. The SM component identifies incorrect and missing field values even when sensors are exchanging a large number of messages as in dynamic scenarios, sensors need to transmit and pass smaller messages compared to SWE standards (Table 3).

#### 5.3.5. Interoperability Analytics Testing of a Flood Citizen Observatory

**Figure 18:**
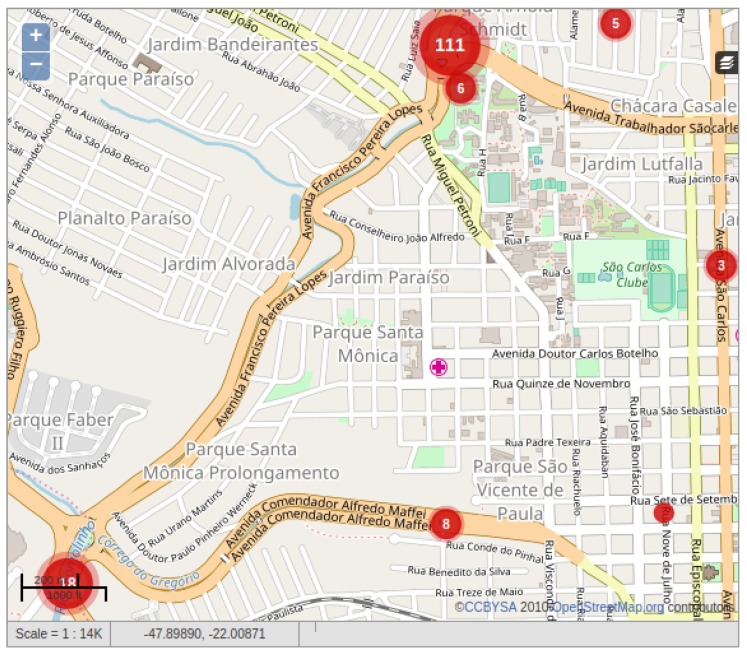
Flood Citizen Observatory.

An interoperability testing aims to check whether one system is compatible on many levels with others. AGORA-DSM enables managing sensor data streams and communicating their data with other systems without prior intimation nor affecting its performance. In addition, it can receive information from crowdsourcing platforms based on the standard on which both systems rely (e.g., SOS). An exemplary crowd-sourcing platform for floods is a citizen observatory where citizens can publish reports containing environmental conditions such as flooded areas descriptions for flood risk management [77]. Based on this, an interoperability analytics evaluation was performed on an instance of Ushahidi (https://www.ushahidi.com/) deployed in the city of São Carlos in Brazil (http://www.agora.icmc.usp.br/enchente/) (see Figure 18). In this platform, each volunter’s observation has its location represented by both latitude and longitude, and a place name. Also, it contains the incident date and a category with the mechanisms used by the voluteer to interpret the observation. That includes scenarios of supervised (e.g., river ruler), semi-supervised (e.g., human shape drawing) and unsupervised (e.g., by deduction) data collections. Additional information are published as a description.

**Figure 19:**
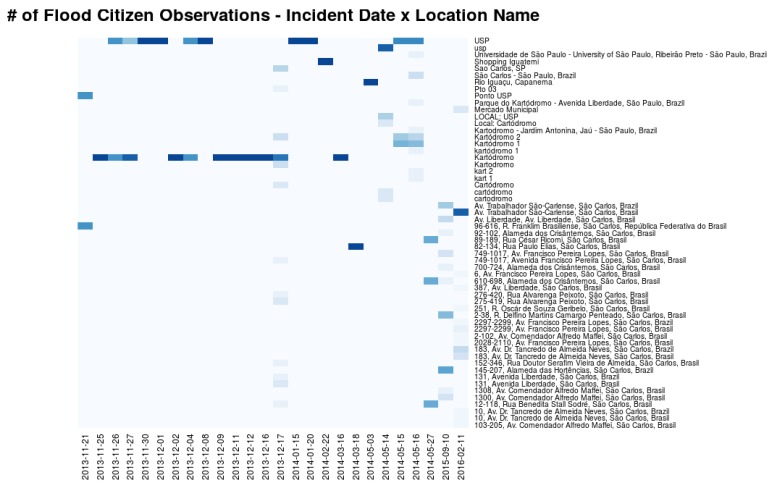
Citizens’ Observations Heatmap.

**Figure 20:**
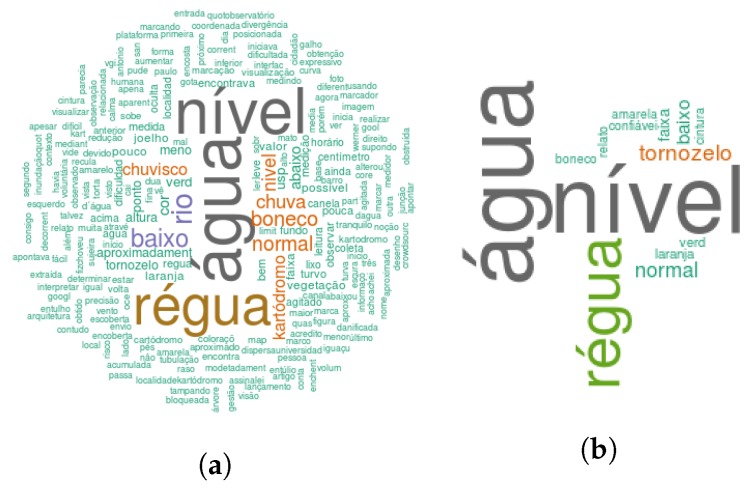
Citizen Observations Description and Category Synthesis. (**a**) Citizen Observations Description WordCloud; (**b**) Citizen Observations Category WordCloud.

Analyzing 194 observations published in the platform, it can be seen through a heat map (see Figure 19) the spelling heterogeneity of the locations provided by the volunteers for every flood occurrence day. As a result, it is hard to analyze the impact and relevance of each flood event by grouping the number of observations and location names since such data is unstructured unlikely sensor data. However, regarding semantics their data is more complete highlighted by visual representation techniques of text data such as tag clouds (see Figure 20). Each category and description fields presented in the volunteer’s observations was processed as a heap of messages containing natural language text. Because of this, it was necessary to clean and wrangle them to unify messy and complex characters into lowercase, removing numbers, removing stop words (a set of irrelevant words) and removing punctuation. The results showed as prominent Portuguese terms: água (water), nível (level), régua (rule), chuva (rain), rio (river), chuvisco (drizzle), boneco (doll), tornozelo (ankle), and kartodromo (a place in the city of São Carlos). These words reflect flood-related terms that the stakeholders agree to make decisions easier. This citizen observatory platform enabled gathering these heterogeneous datasets and encoding them as O&M to being stored at SOS [78], which facilitates its integration with AGORA-DSM.

### 5.4. Analysis of Batch and Stream Management Integration

Spark streaming enables reading from batch and stream input data sources, dividing the data stream into micro batches for processing in memory, thus eliminating network barriers. Spark processes data as a collection of immutable partitioned datasets by means of random sorting [79]. It provides a unified API for structured Java streaming of an unbounded input table to which each new element is appended as a row. The result is obtained from a query applied to the input table and then written as output, a batch-like query called incrementalization in Spark as it augments the dataset. The output retains only the new rows appended to the table. As a result, Sparks enables the middleware to handle continuous sensor data streaming from municipalities across Brazil.

**Figure 21:**
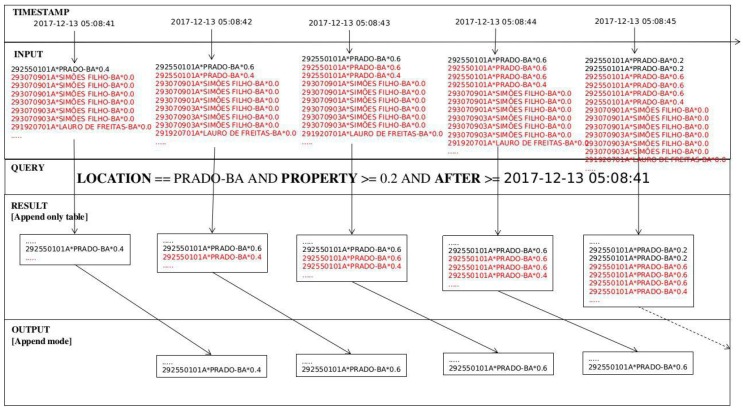
Spark Append Mode.

In Figure 21, a small interval of time can be seen during the second experiment. At that moment (2017-12-13 05:08:41), middleware was gathering sensor data from the state of Bahia. The list shows sensor id, location, and water level, and omits the *publishObservation* field. A query with location field of “PRADO-BA” yielded a water level exceeding 0.2 m and data from 2017-12-13 05:08:41 onwards. The outcome, however, was only stored a second later, a delay due to Spark behaviour.

**Figure 22:**
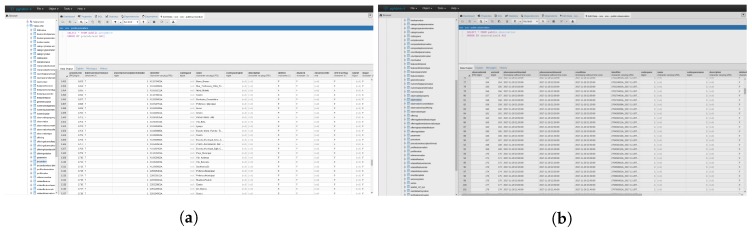
SOS repository. (**a**) Procedure table; (**b**) Observation table.

To connect Spark with a batch source it was necessary to create a single point of entry to encapsulate the functionalities in which the Spark’s streaming driver is used to manage the resources. Subsequently, a database connection was established to enable queries with conditional parameters to the SOS repository (Figure 22). Then the input table was generated so that transformations or actions could be executed. This approach enabled streaming data to be merged with static sensor observations provided by SWE services.

### 5.5. Event Awareness by Sensor Data Filtering

**Figure 23:**
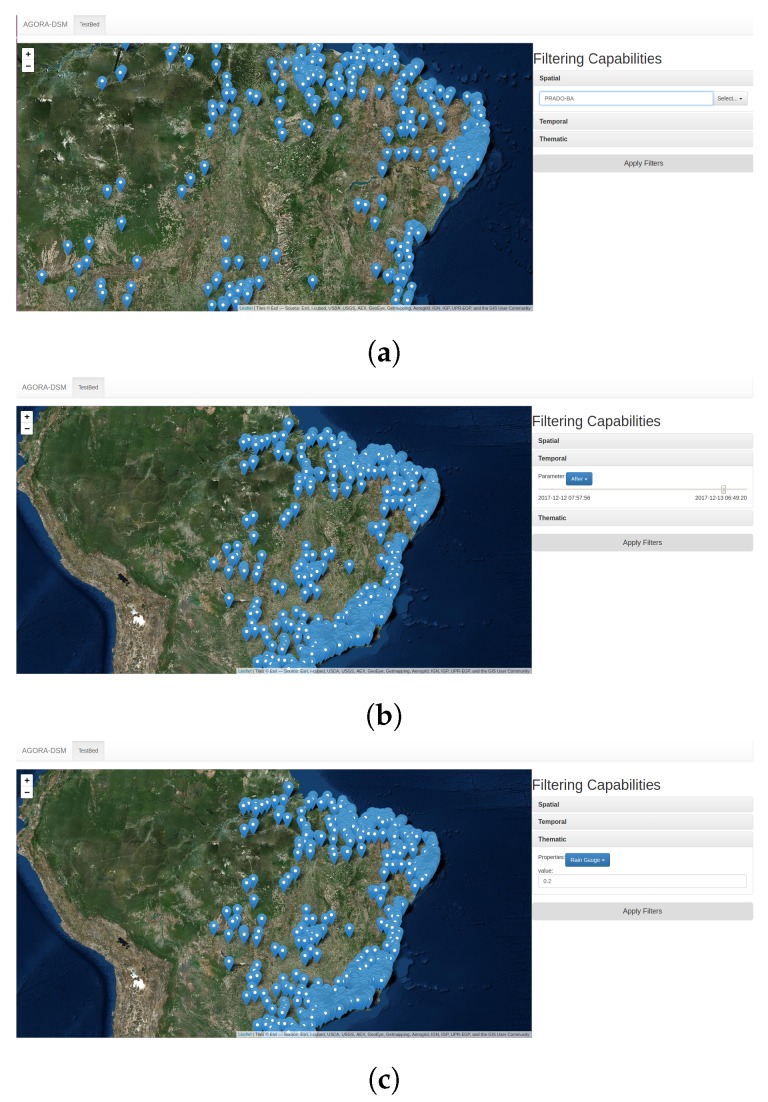
Prototyping Web App—Filtering Capabilities Parameters. (**a**) Spatial Parameters; (**b**) Temporal Parameters; (**c**) Thematic Parameters.

A Web application was implemented to proof the concept of AGORA-DSM, using Leaflet, which simplifies the use of common mapping capabilities and enables developers to incorporate additional features via plug-ins. Several high-performance, lightweight libraries with user-friendly interactive maps, whose features render vector and raster data obtained in different formats, such as GeoJSON and KML. The study used ESRI World Imagery to display sensor observations based on near-real-time queries of sensor data. In that way, one could see a high volume of near-real-time events from sensor data in a more intuitive manner, facilitating the organization of topics with spatial, temporal and thematic parameters (see Figure 23).

**Figure 24:**
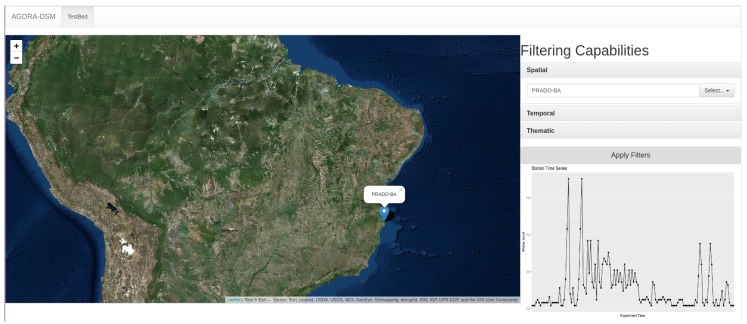
Prototyping Web App—Result Filtering Capabilities.

Subsequently, we applied the parameters described in the previous subsection, inserting a text box with the location name, PRADO-BA, the temporal filtering (2017-12-13 05:08:41) and the property (water level) with its value (0.2), generating a time series that enables one to see historical and near-real-time station behaviour (Figure 24).

## 6. Discussions

This paper proposes an approach to manaing large geosensor data streams involving dynamic scenarios. Its purpose was to determine whether such an approach can provide on-the-fly access, near-real-time publication and event filtering capabilities for heterogeneous geosensor data and, by blending batch and stream approaches, enable large-scale data processing of near-real-time application through interoperable, external and open storage databases. Finally, the middleware was applied to flood risk management scenario in Brazil. The results of a formal, systematic identification, classification, and interpretation of relevant studies to identify existing approaches to integrating sensors into the Sensor Web are described [67]. The search focused on studies that address issues involving scalability, reusability, interoperability, and standardization. From these studies, a set of sensor activities essential to attaining the study’s objective was extracted. Although the systematic mapping revealed the considerable effort required to configure and adapt Sensor Web standards, their service interfaces helped encapsulate sensor functionalities.

### 6.1. Service-Oriented Middleware

Based on their activities, a well-defined, lightweight messaging protocol was designed to mediate between sensors and diverse systems in situations in which it is critical to manage sensor data batches and streams efficiently and communicate without regard to hardware distinctions [22,52]. The protocol facilitates timely decision making in dynamic scenarios. Its message size is about 60 bytes, about 5% and 1% of those JavaScript Object Notation (JSON) and Simple Object Access Protocol (SOAP) SWE standards, respectively. Finally, AGORA-DSM does not require complex pre-processing but works with satellite imagery through an on-demand adapter. AGORA-DSM could be complemented by existing SOS implementations that address satellite imagery as space agencies require a pre-order request following the data search process [15]. The process of requesting, waiting and downloading data can be frustrating at times.

In the study’s four scenarios, the SM component received 292,061, 103,599, 48,166 and 69,643 messages with mean processing times of 3.022692 × 10${}^{-18}$, 5.070575 × 10${}^{-18}$, 2.361595 × 10${}^{-18}$, 2.361595 × 10${}^{-18}$, and 3.174044 × 10${}^{-18}$ and standard deviation values of 2.276596 × 10${}^{-18}$, 2.190448 × 10${}^{-18}$, 2.264901 × 10${}^{-18}$, and 2.886253 × 10${}^{-18}$. The processing time is calculated by measuring the time required to extract, transform and load sensor data into batch and stream management. The study’s time are compatible with the latency required in dynamic scenarios, with even the highest processing times (9.99992 × 10${}^{-18}$, 9.998872 × 10${}^{-18}$, 9.99725 × 10${}^{-18}$, 9.999931 × 10${}^{-18}$) sufficient to enable adequate management. The SM component resource utilization was stable with acceptable median CPU use (CPU: 85.48745; memory: 8.711755), and the number of requests per time analyzed varied little after initial requests, as reflected in the mode values (3; 1; 1; 1).

### 6.2. Joining Batch and Stream Processing

As SWE standards were taken into account, AGORA-DSM can add interoperability to systems [34,35,36,37,38,39,40,42,43,44] that manage geosensors with their own means and help manage distinct sensor data format and flow in dynamic scenarios [80] with control and software components function in an interoperable manner . These re-usable components were tested in 4 ad hoc scenarios. It should be noted that the study’s scenarios are complex and dynamic, and AGORA-DSM’s objective is to mask this complexity [53,54] by managing heterogeneous geosensor (stationary and mobile geosensors, and citizen sensor) messages with low latency in a scalable manner. As noted, this approach complements structured data provided by in situ sensors [21] with unstructured, asymmetric data streams. Integrating heterogeneous data sources, such as authoritative and volunteered data, to assess on-the-ground conditions is not a new concept [6,13], an the intent here is to facilitate their access via filtering capabilities.

Combining a batch mechanism in a push-based manner with a stream processing engine not only enables content-based filtering based on applications subscriptions [61] but can provide data-stream filtering. StrM component works with interactive queries and unified interfaces incrementally. BM and StrM are integrated to work with event-stream processing, with StrM designed to work on a server side event-stream filtering unlike described in BM specifications. StrM also enables management of failures and keeps application synchronized with BM components. With this in mind, EM was structured on the concept of data filtering and discovering to enhance event awareness in dynamic scenarios.

### 6.3. Leassons Learned from Flood Risk Management

**Figure 25:**
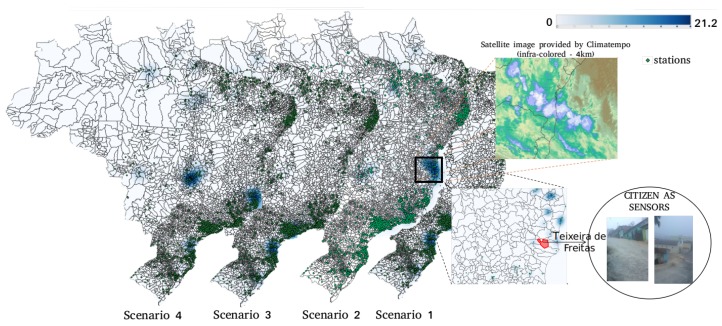
Brazilian scenarios of flash floods.

Updated data on river conditions is critical to inform decision-making in flood risk management, but several technical factors could impede traditional sensor networks from functioning properly. Developing countries such as Brazil, where flash floods from heavy rains and overflowing waterways are frequent, need efficient, effective instruments to mitigate the risk of human casualties and flood damage. The findings reported herein of fast processing times during critical periods evidence the potential of AGORA-DSM to yield accurate, timely warnings [81]. During the study (see Figure 25), situations occurred in which sensors were constantly measuring high values, stopped sending their messages and reported incorrect values, as which in turn may reflect on the unavailability and misleading of information sharing. This could arise as a result of their position on the river. Heavy rains might affect the infrastructure, including cellphone services and Wi-Fi.

Local citizens may complement regional sensor data [6,12], resulting in no previously assigned risk areas being in the decision-makers’ view [82,83,84]. An example occurred in Teixeira de Freitas, Bahia, where CEMADEN had no stationary sensors deployed, but citizens recorded how critical situation was. Although citizen sensors present some limitations in the quantity of their data, sometimes little information is enough. The value of mobile sensors, such as UAVs, have also reported in previous works [22].

Satellite imagery could improve decision making by providing an overview of large areas, showing, for instance, how clouds are moving in risk areas. With this type of data, prompt action could be taken to minimize damages caused by natural disasters. In the study, satellite confirmed the data on a heat map of water levels generated by stationary sensors. The study also highlighted significant issues like communication overhead in real-life scenarios, such as disaster management, e-agriculture, and smart cities, that simulations cannot [62]. As the authors’ research has shown, an increasing number of timely application requests from non data-centric architecture can be generated from ubiquitous, spatial, temporal, and thematic geosensor discovery. As a consequence, they intend to share sensor information for regional areas in dynamic domains, such as disaster management, rather than local ones with stationary domain applications [63,85]. This includes such other domains such as e-agriculture, smart cities and crime monitoring. To undertake this research will require the development of an evaluative method to determine the generality of the middleware, a task beyond the scope of this study.

## 7. Conclusions

Sensor networks are used in environmental monitoring to provide large amount of data in near real-time. Stationary sensors measure water levels, temperature, and pressure, while mobile sensors measure areas where humans cannot safely. Nevertheless residents of at risk areas have contributed to environmental monitoring complementing the coverage of traditional sensor networks.

The role of heteogeneous data providers is challenging when combed in dynamic scenarios. Fowarding their data to applications through a filtering process requires a user-friendly interface. The approach proposed in this study affords interoperable mechanisms to integrate heterogeneous sensors and facilitate access to, publication of, and filtering of their data in on line in near real-time. To this end, the study conducted an application case study of flood risk management.

Its results evidence that a simple protocol can synthesize sensor activities and enhance interoperability among sensor networks and batch and stream components. Since the AGORA-DSM is readily translated and interpreted, its latency is minimal, which is crucial in near-real-time application. The number of sensors was varied in the study’s tests to better assess the protocol’s scalability. Issues arise when sensors experience hardware limitations or are deployed in locations devoid of stable Internet connections. Consistent platforms such as SWE implementations and Spark were used for batch and stream operations, essential to near-real-time large scale data-stream management. Further research should focus on security constraints and machine learning libraries. Application of AGORA-DSM scenarios and organizational millieus should be conducted to assess its generality and expand the knowledge base.

References

## Figures and Tables

**Table 1:** Summary of the approaches and their main functionalities.

SWE	Main Functionalities													Research Group—References
	1	2	3	4	5	6	7	8	9	10	11	12	13	
√	×	×	×	×	×	×	×	×	×	×	×		×	[52,53,68,69]
														[21,54,55,56,61]
	×		×	×	×	×		×		×				[70,71,72,73]
	×		×	×	×							×		[49]
√	×	×	×	×	×	×	×	×	×	×				[50]
√	×		×			×	×	×		×				[45]
√	×		×	×	×	×	×	×	×	×		×	×	[47]
√	×		×	×	×	×		×		×			×	[48]
	×	×			×	×	×	×	×	×				[16,37,38]
√	×	×	×	×	×	×		×		×	×		×	[18]
√	×		×			×	×	×	×	×			×	[14]
							×	×		×		×		[74]
			×		×		×			×		×	×	[75]
						×		×		×				[76]
	×	×			×	×	×	×	×	×				[36]

* Functionalities. 1. Sensor Registration; 2. Selection of Specific Sensors; 3. Data Publication; 4. Observation Repository; 5. Service Registration; 6. Access to Sensor Metadata; 7. Sensor Tasking; 8. Access to Sensor Historical Data; 9. Sensor Subscription; 10. Access to Sensor Near-Real-Time Data; 11. Semantic Correspondence between Sensors and Services; 12. Service Subscription; 13. Access to Service Metadata.

**Table 2:** Message Protocol.

Message	Syntax
**Register Sensor**	registerSensor*sensorID*observedProperty
**Publish Observation**	publishObservation*sensorID*timestamp*location*value
**Start Monitoring**	startMonitoring*sensorID*timestamp*location
**Stop Monitoring**	stopMonitoring*sensorID*timestamp*location
**Sleep Monitoring**	sleepMonitoring*sensorID*timestamp*location
**Wake up Monitoring**	wakeUpMonitoring*sensorID*timestamp*location
**Current Position**	currentPosition*sensorID*timestamp*location

**Table 3:** Size in Bytes Comparison.

Message Protocol		SWE Standard			
**TEXT**		**JSON**		**SOAP**	
**Operation**	**Size**	**Operation**	**Size**	**Operation**	**Size**
register sensor	42	DescribeSensor	161	DescribeSensor	866
(registerSensor*sensorID*observerdProperty)		InsertSensor	4433	InsertSensor	8325
publish observation	60	GetObservationById	125	GetObservationById	680
(PublishObservation*sensorID*timeStamp*location*value)		InsertObservation	1084	InsertObservation_Measurement	2462
stop monitoring	41	DescribeSensor	161	DescribeSensor	866
(StopMonitoring*sensorID*timeStamp*location)		UpdateSensorDescription	2922	UpdateSensorDescription	4348
start monitoring	49	DescribeSensor	161	DescribeSensor	866
(StartMonitoring*sensorID*timeStamp*location)		UpdateSensorDescription	2922	UpdateSensorDescription	4348
sleep monitoring	44	DescribeSensor	161	DescribeSensor	866
(SleepMonitoring*sensorID*timeStamp*location)		UpdateSensorDescription	2922	UpdateSensorDescription	4348
wake up monitoring	50	DescribeSensor	161	DescribeSensor	866
(WakeUpMonitoring*sensorID*timeStamp*location)		UpdateSensorDescription	2922	UpdateSensorDescription	4348
current position	49	DescribeSensor	161	DescribeSensor	866
(CurrentPosition*sensorID*timeStamp*location)		UpdateSensorDescription	2922	UpdateSensorDescription	4348

## Supplementary Materials

All data created during this research are openly available from the University of Warwick data archive at http://wrap.warwick.ac.uk/102340.
